# Discovery of Myeloid-Derived Suppressor Cell-Specific Metabolism by Metabolomic and Lipidomic Profiling

**DOI:** 10.3390/metabo13040477

**Published:** 2023-03-27

**Authors:** Jisu Kim, Hwanhui Lee, Hyung-Kyoon Choi, Hyeyoung Min

**Affiliations:** College of Pharmacy, Chung-Ang University, Seoul 06974, Republic of Korea

**Keywords:** myeloid-derived suppressor cell, metabolomics, lipidomics

## Abstract

The endogenous factors that control the differentiation of myeloid-derived suppressor cells (MDSCs) are not yet fully understood. The purpose of this study was to find MDSC-specific biomolecules through comprehensive metabolomic and lipidomic profiling of MDSCs from tumor-bearing mice and to discover potential therapeutic targets for MDSCs. Partial least squares discriminant analysis was performed on the metabolomic and lipidomic profiles. The results showed that inputs for the serine, glycine, and one-carbon pathway and putrescine are increased in bone marrow (BM) MDSC compared to normal BM cells. Splenic MDSC showed an increased phosphatidylcholine to phosphatidylethanolamine ratio and less de novo lipogenesis products, despite increased glucose concentration. Furthermore, tryptophan was found to be at the lowest concentration in splenic MDSC. In particular, it was found that the concentration of glucose in splenic MDSC was significantly increased, while that of glucose 6-phosphate was not changed. Among the proteins involved in glucose metabolism, GLUT1 was overexpressed during MDSC differentiation but decreased through the normal maturation process. In conclusion, high glucose concentration was found to be an MDSC-specific feature, and it was attributed to GLUT1 overexpression. These results will help to develop new therapeutic targets for MDSCs.

## 1. Introduction

Myeloid-derived suppressor cells (MDSCs) are heterogeneous immature myeloid cells that can suppress the immune response by inhibiting the function of immune cells such as dendritic cells, natural killer cells, and T cells. Under pathological conditions, including inflammatory diseases, infection, autoimmune disorders, and cancer, MDSCs are produced in the bone marrow (BM) and then migrate to peripheral lymphoid organs and tumor sites [1]. In mice, the phenotype of MDSCs is well-established as CD11b^+^Gr-1^+^. It can be further sub-classified into two subtypes: CD11b^+^Ly6G^−^Ly6C^hi^ monocytic MDSCs (mMDSCs) and CD11b^+^Ly6G^+^Ly6C^lo^ polymorphonuclear MDSCs (pmnMDSCs). In humans, mMDSCs are defined as CD11b^+^CD33^+^HLADR^−/lo^CD66b^−^CD15^−^CD14^+^, while pmnMDSCs are defined as CD11b^+^CD33^+/lo^CD66b^+^CD15^+^CD14^−^ with a low density. However, the phenotype and function of human MDSCs are not well characterized, and further research on these aspects is required [2].

Under normal circumstances, 2–3% of splenocytes in C57BL/6 mice are comprised CD11b^+^Gr-1^+^ cells. However, this proportion increases significantly to 25% of splenocytes in tumor-inoculated mice [3]. MDSCs inhibit T cells directly by engaging programmed cell death ligand 1 and programmed cell death protein 1 and indirectly by secreting reactive oxygen species, immunosuppressive transforming growth factor-β and indolamin-2,3-dioxygenase (IDO), which exhausts tryptophan, an essential amino acid for the T cell immune response [4]. As a result, MDSCs induce T cell anergy and inhibit the cytotoxic activity of CD8^+^ cytotoxic T cells. The activity of CD4^+^ effector T cells is also inhibited, while the immunosuppressive activity of regulatory T cells is increased by MDSCs. In addition to T cells, MDSCs also inhibit other myeloid cells, such as dendritic cells, macrophages, and natural killer cells, by secreting immunosuppressive cytokine IL-10 [5].

MDSCs enable cancer cells to evade immune surveillance, and a high level of MDSCs is strongly associated with a poor prognosis in cancer patients [6]. During anti-tumor therapy with chimeric antigen receptor (CAR)-T cells, MDSCs residing in the tumor microenvironment suppressed the anti-tumor effect of CAR-T cells and became a therapeutic obstacle, particularly in solid tumors [7]. Current approaches to treating MDSCs include eliminating them, blocking their recruitment, reducing their immunosuppression, and inducing their terminal differentiation [8].

Cells generate adenosine triphosphate (ATP) by a molecular breakdown process known as catabolism to obtain energy. In cellular metabolism, nutrients are metabolized into other substances, and each step is strictly regulated by specific enzymes. The metabolic processes in cells are intricate and interconnected [9], and attempts have been made to treat diseases by targeting metabolic pathways [10]. For example, in cancer cells, uncontrolled proliferation creates a high demand for energy and cellular components, and particular metabolic pathways are excessively activated. Therefore, research focuses on therapeutic strategies to inhibit highly upregulated metabolism in cancer cells. Specifically, promising candidates targeting glucose metabolism are being investigated [11].

Metabolomic analysis has emerged as a powerful research tool to understand the underlying mechanisms of unique features in various cell types [12,13]. Umemura et al. analyzed the metabolites of CD11b^+^ splenocytes and tumor-infiltrated macrophages, which are cell types related to MDSCs, by capillary electrophoresis-time-of-flight-mass spectrometry [14]. However, the metabolomic profile of MDSCs has not yet been studied thoroughly. Furthermore, the composition and abundance of lipids, which are important in membrane function and cell signaling, have not been investigated in MDSCs. Therefore, here we analyzed the metabolomic and lipidomic profiles of MDSCs to find an MDSC-specific metabolism that can be used as a therapeutic target.

## 2. Materials and Methods

### 2.1. Mouse Model

Male C57BL/6 mice (5 to 6 weeks old) were purchased from Raonbio (Yongin-si, Gyeonggi-do, Republic of Korea). The mice used in the experiments were housed in a specific pathogen-free environment with temperature control and a 12:12 h light/dark cycle. All mice had ad libitum access to food and tap water during the experiments. This study was approved by the Institutional Animal Care and Use Committee of Chung-Ang University (approval number 202000092).

### 2.2. Preparation of In Vivo-Generated MDSCs by Cell Sorting

On day 0, C57BL/6 mice were inoculated subcutaneously at the right flank with 1 × 10^5^ EL-4 mouse lymphoma cells (KCLB No. 40039, Korean Cell Line Bank, Seoul, Republic of Korea). At day 21, the femur, tibia, and spleen were harvested from the tumor-inoculated mice and age-matched normal mice. BM cells were flushed from the tibia and femur with phosphate-buffered saline (PBS). After lysis of red blood cells (RBC) by ACK lysis buffer (10 mM KHCO_3_, 0.1 mM Na_2_EDTA, 0.15 M NH_4_Cl, pH 7.2, Sigma-Aldrich, St. Louis, MO, USA), cells were centrifuged, resuspended in PBS, and passed through a 70 µm cell strainer tube.

To increase the concentration of CD11b^+^ cells in splenocytes before sorting, the splenocytes were resuspended in MACS buffer (PBS with 0.5% bovine serum albumin, 2 mM EDTA, pH 8.0) and anti-CD11b microbeads were added (Miltenyi Biotec, Bergisch Gladbach, NRW, Germany). CD11b^+^ cells were positively selected using a magnetic MidiMACS Separator (Miltenyi Biotec). The isolated CD11b^+^ splenocytes and BM cells were then stained with phycoerythrin (PE) anti-Gr-1 monoclonal antibody (mAb, clone RB6-8C5) and fluorescein isothiocyanate (FITC) anti-CD11b mAb (clone M1/70, eBioscience, Waltham, MA, USA). The stained cells were sorted using a FACS AriaII (BD Biosciences, Franklin Lakes, NJ, USA).

The sorted cells were centrifuged for 2 min at 1000× *g*, after which the cell pellets were stored at −80 °C for later analysis. A modified Folch procedure was used to extract lipids and metabolites from the freeze-dried cells, as described by Kim et al. [12].

### 2.3. GC-MS and NanoESI-MS Analyses

The metabolite and lipid analyses of the extracted samples of MDSCs were conducted using gas chromatography-mass spectrometry (GC-MS) and nanoelectrospray ionization-mass spectrometry (nanoESI-MS) according to a previously described method [12].

### 2.4. MDSC Differentiation In Vitro

The mice’s femurs and tibias were harvested, and BM cells were flushed out with PBS. After RBC lysis, the cells were centrifuged and resuspended in complete RPMI1640 media containing fetal bovine serum (Welgene, Gyeongsan-si, Gyeongsangbuk-do, Korea), l-glutamine (Corning, Corning, NY, USA), MEM non-essential amino acids (Corning), sodium pyruvate (Corning), HEPES (Corning), penicillin/streptomycin (Gibco, Grand Island, NY, USA), and 2-mercaptoethanol (Sigma-Aldrich). In order to promote differentiation of MDSCs, BM cells were cultured with 40 ng/mL of IL-6 and 40 ng/mL of GM-CSF at 37 °C in a humidified 5% CO_2_ atmosphere for 4 days. MDSC maturation was induced by treating with 2 μM of all-trans retinoic acid (ATRA) for 4 days during MDSC differentiation, as previously described [15].

### 2.5. Quantitative Real-Time PCR

Cells were lysed by RNAiso (Takara, Otsu, Shiga, Japan). The total RNA was extracted and then reverse-transcribed into complementary DNA using Easyscript Reverse Transcriptase (TransGen Biotech, Beijing, China). Quantitative real-time PCR was conducted using TOPreal qPCR 2X PreMIX (Enzynomics, Daejeon, Republic of Korea) with a CFX Connect Real-Time PCR Detection System (Bio-Rad, Hercules, CA, USA). The mRNA expression level was normalized by *Actb*. Primers with the following sequences were used: *Hk1* (sense, CGGAATGGGGAGCCTTTGG; antisense, GCCTTCCTTATCCGTTTCAAT), *Hk2* (sense, GGGCATGAAGGGCGTGTCCC; antisense, TCTTCACCCTCGCAGCCGGA), *Hk3* (sense, CTGAGTCAAGGCTGTATCCTCC; antisense, TGCACCAGTTCAGCATCTGAGG), *G6pc3* (sense, AGCAGGTAGCCGCTTCATTT; antisense, AGCCGGTTCTGTAGTGCTTC), *Glut1* (sense, TCAACACGGCCTTCACTG; antisense, CACGATGCTCAGATAGGACATC), *Glut3* (sense, TTCTGGTCGGAATGCTCTTC; antisense, AATGTCCTCGAAAGTCCTGC), *Phgdh* (sense, CCTCCTTTGGTGTTCAGCAGCT; antisense, CGCACACCTTTCTTGCACTGAG), *Actb* (sense, ACCCACACTGTGCCCATCTAC; antisense, GCCATCTCCTGCTCGAAGTC).

### 2.6. Flow Cytometry

Cells were fixed and permeabilized with Foxp3/Transcription Factor Staining Buffer set (eBioscience). Cells were stained with mAbs and then analyzed using a FACSCalibur (BD Biosciences). Antibodies used were anti-GLUT1 mAb (clone SPM498, eBioscience), Alexa Fluor 488 anti-mouse IgG (H+L), F(ab’)_2_ fragment (Cell Signaling, Danvers, MA, USA), Peridinin-Chlorophyll-Protein (PerCP) anti-CD11b mAb (clone M1/70, BioLegend, San Diego, CA, USA), FITC anti-Ki-67 mAb (clone SolA15, eBioscience), and PE anti-Gr-1 mAb (clone RB6-8C5, eBioscience).

### 2.7. Statistical Analysis

Raw data files from the lipid analysis (*.raw) were converted into *.mzXML format using Proteo Wizard MSConvert software (v.3.0). The metabolite and lipid mass spectra were transformed into the resulting data set using Expressionist^®^ MSX software (v.2013.0.39, Genedata, Basel, Switzerland) for further data processing. The data obtained from MDSCs were divided by the peak intensity of the internal standard and the total protein content for normalization. Significant differences in compounds were identified by a Mann–Whitney U test using SPSS software (v.26, IBM, Somers, NY, USA). All data were mean-centered and pretreated to Pareto scaling for partial least squares discriminant analysis (PLS-DA), which was performed using SIMCA-P software (v.15.0.2, Sartorius Stedim Data Analytics AB, Umeå, Sweden). Cross-validations and permutation tests (100 times) were performed to evaluate the validity of the PLS-DA models.

Significant differences in mRNA and protein expression levels were identified by a one-way ANOVA followed by Tukey’s post-hoc test or a two-way ANOVA followed by the Bonferroni post-hoc test or Student’s *t*-test using Prism 5 (v.5.03, GraphPad Software, San Diego, CA, USA).

## 3. Results

### 3.1. Comprehensive Metabolomic and Lipidomic Profiling of Mouse BM Cells and MDSCs

As MDSCs are composed of heterogeneous and immature cells of the myeloid lineage, many types of cells can be used as controls [16,17,18]. To determine the differences between non-immunosuppressive normal myeloid cells and immunosuppressive MDSCs, mature MDSCs and myeloid precursor cells from the BM of tumor-bearing mice and normal mice were selected for metabolomic and lipidomic analyses. The CD11b^+^Gr-1^+^ BM cells from normal mice (normal BM) have an identical surface marker to that of MDSCs and were used as controls. CD11b^+^Gr-1^+^ cells from the BM of tumor-bearing mice (BM-MDSCs) were chosen to represent differentiating MDSCs, while CD11b^+^Gr-1^+^ cells from the spleen of tumor-bearing mice (SPL-MDSCs) were selected as mature MDSCs. Each cell type was sorted using a flow cytometer, and the purity of each sorted cell type was >98.0% (Figure S1).

**Figure 1:**
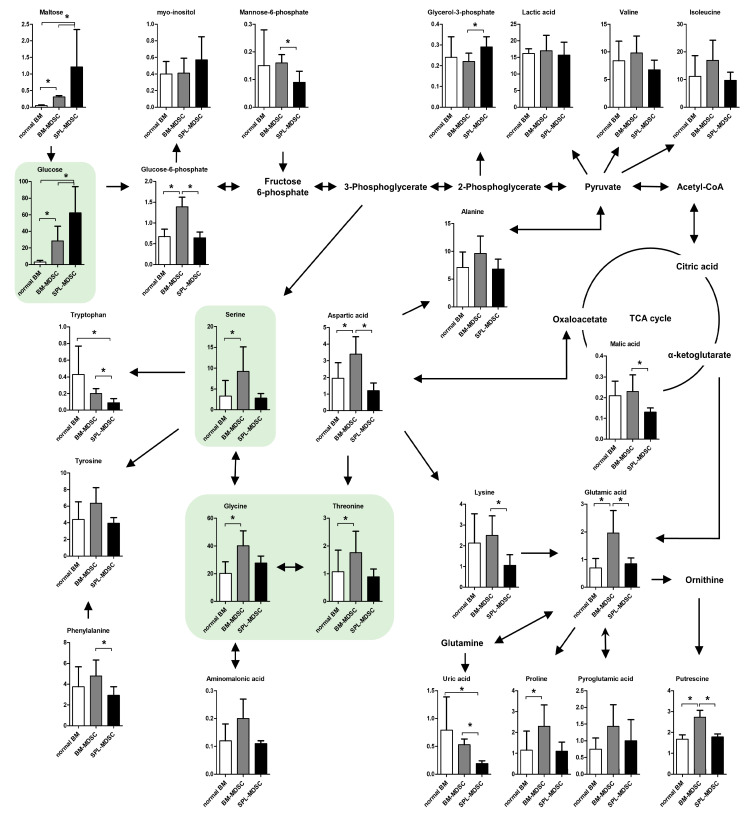
Comparative metabolomic profiles in BM cells and MDSCs. The proposed metabolic pathway is presented based on data in the KEGG database (http://www.genome.jp/kegg/, accessed on 4 November 2022). Each value represents the mean ± standard deviation (SD) (*n* = 6, three biological and two experimental replicates). *, *p* < 0.05. Green boxes indicate SGOC-pathway-related metabolites. The *p* values were obtained by a Mann–Whitney U test.

Comprehensive metabolomic profiling of BM cells and MDSCs was conducted using GC-MS, and twenty-five metabolites were identified: fourteen amino acids, six carbohydrates, three organic acids, putrescine, and uric acid (UA) (Figure 1).

Among the amino acids, aspartic acid, glutamic acid, glycine, proline, serine, and threonine were increased in BM-MDSCs compared to normal BM. Aspartic acid, glutamic acid, lysine, and phenylalanine were decreased in SPL-MDSC compared to BM-MDSC. Tryptophan and UA concentrations were the lowest in SPL-MDSCs. Putrescine, metabolized from arginine, presented the highest concentration in BM-MDSCs.

Among the carbohydrates, glucose, glucose-6-phosphate (G6P), and maltose were increased in BM-MDSCs compared to normal BM. Glucose and maltose were increased in SPL-MDSCs compared to normal BM. SPL-MDSCs showed higher concentrations of glucose, glycerol-3-phosphate, and maltose compared to BM-MDSCs, while G6P, mannose-6-phosphate, and malic acid were decreased.

**Figure 2:**
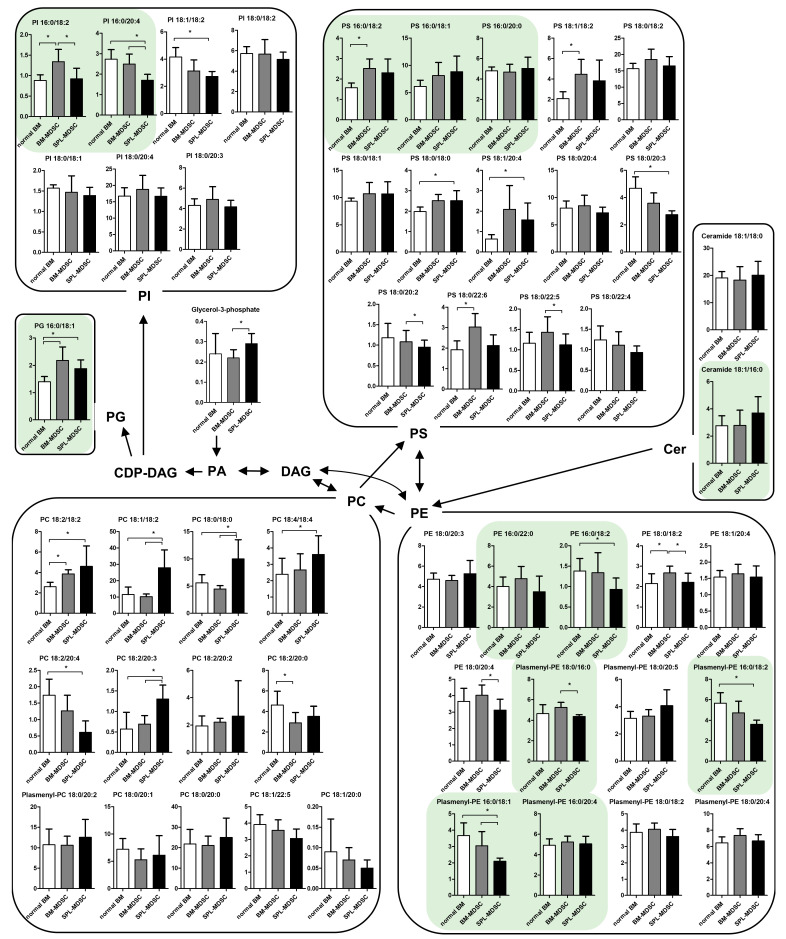
Comparative lipidomic profiles in BM cells and MDSCs. The proposed metabolic pathway is presented based on data in the KEGG database (http://www.genome.jp/kegg/, accessed on 4 November 2022). Each value represents the mean ± standard deviation (SD) (*n* = 6, three biological and two experimental replicates). *, *p* < 0.05. Green boxes indicate lipids containing palmitic acid (C16). The *p* values were obtained by a Mann–Whitney U test.

For comprehensive lipidomic profiling of BM cells and MDSCs, nanoESI-MS was conducted, and fifty lipids were identified: twelve phosphatidylcholines (PCs), one plasmenyl-PC, six phosphatidylethanolamines (PEs), seven plasmenyl-PEs, two ceramides, one phosphatidylglycerol (PG), fourteen phosphatidylserines (PS), and seven phosphatidylinositols (PI) (Figure 2).

Among all the lipids, seven were increased, while one was decreased in BM-MDSCs compared to normal BM. In SPL-MDSC, eight lipids were increased, and seven were decreased compared to normal BM. Compared to BM-MDSCs, in SPL-MDSCs, three lipids were increased, and eight were decreased. PCs and PEs showed remarkable changes in SPL-MDSCs. Compared to normal BM, five PCs were increased while one was decreased in SPL-MDSCs. Compared to BM-MDSCs, three PCs were increased in SPL-MDSCs. Two PEs and two plasmenyl-PEs were decreased in SPL-MDSCs.

### 3.2. Multivariate Statistical Analysis of Metabolomic and Lipidomic Profiles of BM Cells and MDSCs

**Figure 3:**
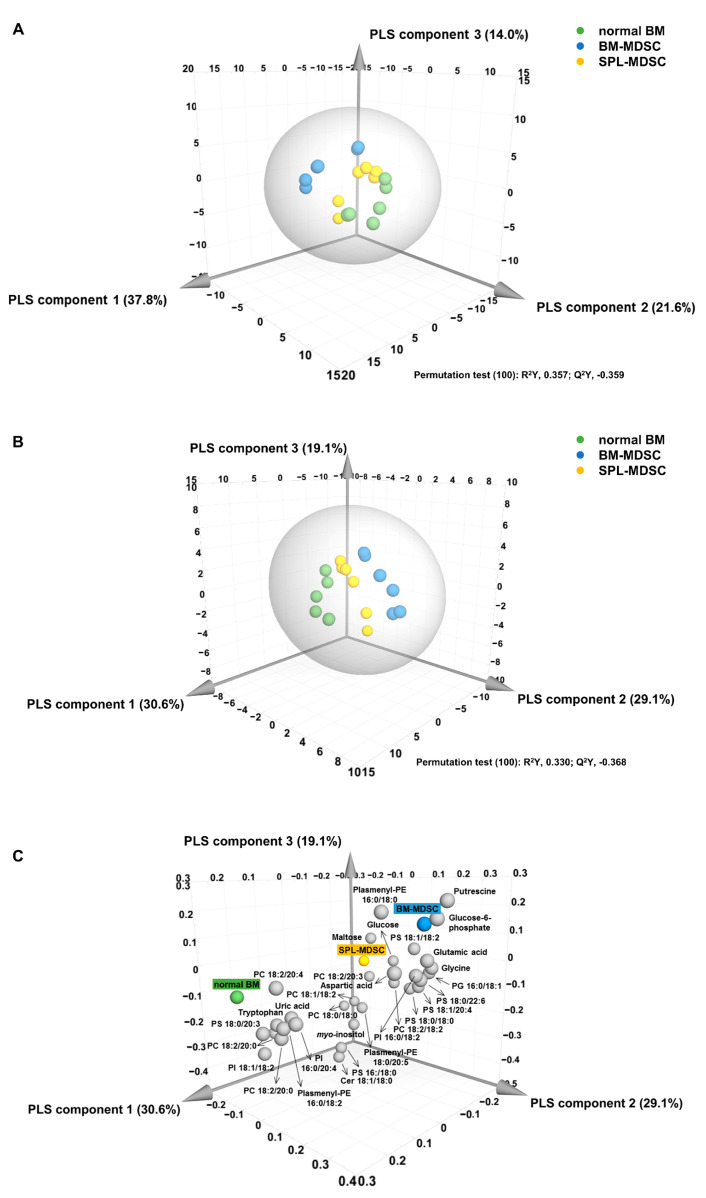
PLS-DA analysis. PLS-DA analysis from metabolomic and lipidomic profiling data for BM cells and MDSCs. (**A**) PLS-DA score plot. (**B**) Regenerated PLS-DA score plot based on components with high VIP values over 1.0. (**C**) PLS-DA loading plot derived from metabolomic and lipidomic profiling data for normal BM, BM-MDSCs, and SPL-MDSCs.

To compare the three groups of cells, PLS-DA was applied to the measured lipidomic and metabolomic profiles. As shown in Figure 3A, each group was clearly distinguishable on the PLS-DA score plot. The optimized PLS-DA model was generated based on the PLS-DA model using 32 compounds with a variable influence on projection (VIP) value of 1.0 or higher, which has a large influence on the separation of groups (Figure 3B) (Table 1).

Permutation tests were performed to assess the validity of the models. The R^2^Y intercept values in our study were 0.357 (Figure 3A) and 0.330 (Figure 3B), and the Q^2^Y intercept values were −0.359 (Figure 3A) and −0.368 (Figure 3B). These values satisfied the criteria of R^2^Y intercept < 0.4 and Q^2^Y intercept < 0.05, confirming the validity of the models [19]. The specific substances related to each group were identifiable by using the PLS-DA loading plot corresponding to the optimized PLS-DA score plot (Figure 3C).

The data indicated that, in normal BM, PS 18:0/20:3, PC 18:2/20:4, UA, and tryptophan were relatively elevated. In BM-MDSCs, plasmenyl-PE 16:0/18:0, G6P, and putrescine were relatively elevated. In SPL-MDSCs, PC 18:2/20:3, maltose, and glucose were relatively elevated.

### 3.3. Investigation of Glucose-6 Phosphate Converting Enzymes

**Figure 4:**
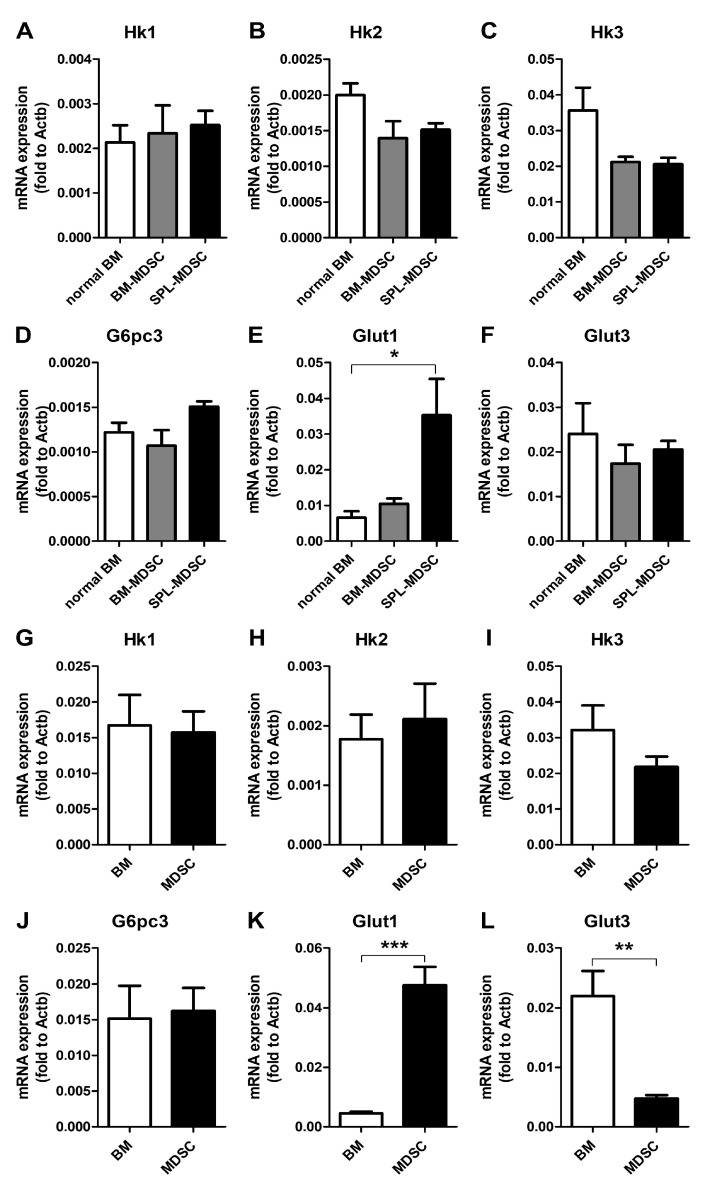
mRNA expression levels of genes related to glucose concentration in MDSCs. (**A**–**F**) Cells were harvested from normal or tumor-engrafted mice. (**G**–**L**) MDSCs were differentiated from mouse BM cells in vitro. The mRNA expression levels were measured by qRT-PCR and normalized by *Actb*. Data shown are expressed as mean ± SEM of three replicates with similar results. *, *p* < 0.05; **, *p* < 0.01; ***, *p* < 0.001. (**A**–**F**) The *p* values were obtained by a one-way ANOVA followed by Tukey’s post-hoc test. (**G**–**L**) The *p* values were obtained by Student’s *t*-test.

Figure 1 shows that the intracellular glucose/G6P ratio was tremendously increased in SPL-MDSCs. The glucose/G6P ratios in normal BM, BM-MDSCs, and SPL-MDSCs were 4.63, 20.46, and 97.53, respectively. Three groups of proteins are involved in converting glucose into G6P: hexokinases (HKs), glucose-6-phosphatases (G6Pases), and glucose transporters (GLUTs). qRT-PCR was performed to measure the mRNA levels of genes involved in glucose phosphorylation. The results revealed that *Glut1* was significantly increased in SPL-MDSCs compared to normal BM cells, while no significant difference was found in *Hk1*, *Hk2*, *Hk3*, *G6pc3* (G6Pase Catalytic Subunit 3), and *Glut3* genes (Figure 4E). In addition, we found that *Glut1* mRNA expression was increased as BM cells differentiated into MDSCs upon treatment with GM-CSF and IL-6 in vitro (Figure 4K).

**Figure 5:**
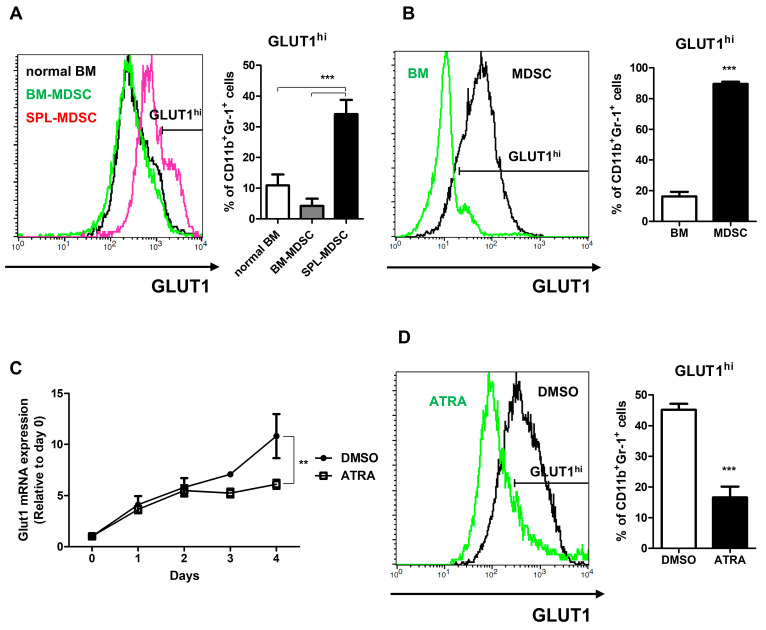
GLUT1 expression level in MDSCs. (**A**) Cells were harvested from normal or tumor-engrafted mice. (**B**) MDSCs were differentiated from mouse BM cells in vitro. GLUT1 expression levels of CD11b^+^Gr-1^+^ cells were measured by flow cytometer. (**C**,**D**) MDSCs were differentiated into MDSCs with or without ATRA treatment. (**C**) Samples were harvested each day, and the *Glut1* mRNA expression levels were assessed by qRT-PCR. (**D**) On day 4, samples were harvested, and the GLUT1 expression levels were measured by flow cytometer. Data shown are expressed as mean ± SD of three replicates with similar results. **, *p* < 0.01; ***, *p* < 0.001. (**A**) The *p* values were obtained by a one-way ANOVA followed by Tukey’s post-hoc test. (**B**,**D**) The *p* values were obtained by Student’s *t*-test. (**C**) The *p* values were obtained by a two-way ANOVA followed by a Bonferroni post-hoc test.

Furthermore, flow cytometry data showed that GLUT1 was overexpressed in SPL-MDSCs (Figure 5A) and in vitro-induced MDSCs (Figure 5B). Next, to confirm that overexpression of GLUT1 is a unique feature of MDSCs, in vitro-induced MDSCs were terminally matured, and the GLUT1 expression level was assessed. In order to generate mature MDSCs, MDSCs were treated with ATRA, which diminishes MDSCs by terminal maturation [20]. The data showed that the expression of GLUT1 mRNA and protein were both decreased by ATRA treatment in vitro (Figure 5C,D). The GLUT1^hi^ ratio in in vitro-induced MDSCs was 45.2% in the DMSO-treated group and 16.6% in the ATRA-treated group.

## 4. Discussion

Intracellular metabolism regulates cellular fate and differentiation [21]. In the case of BM cells, a high-fat diet induces differentiation of BM progenitor cells into adipocytes, a process that can be prevented in vitro by treatment with oligomycin, an inhibitor of oxidative phosphorylation (OXPHOS) [22]. Since MDSCs do not complete normal differentiation and remain in an immature state, this study was conducted to investigate if metabolic alterations play a role in the incomplete differentiation of myeloid cells and generations of MDSCs.

In the serine, glycine, and one-carbon (SGOC) pathway, nutrient sources such as glucose, glycine, serine, and threonine act as inputs, while glutathione buffering oxidative stress and diverse biosynthetic precursors are outputs. Recent studies have shown that increased SGOC metabolism is a feature of rapidly proliferating cells such as cancer cells, and SGOC has been proposed as a therapeutic target of cancer [23,24,25,26]. Our experiment revealed that glucose, serine, glycine, and threonine, which are the inputs of the SGOC pathway, are all increased in BM-MDSCs compared to normal BM. These results suggest that, among the two immature and proliferating cell types, BM-MDSCs with abundant metabolic precursors can activate the SGOC pathway more potently compared to normal BM. However, abundant inputs are not enough to justify the activation of the SGOC pathway. To measure SGOC pathway activity, we used qRT-PCR to assess the expression levels of PHGDH, which mediates the initial rate-limiting step in the SGOC pathway [27]. The results showed that the expression level of *Phgdh* mRNA was higher in BM-MDSCs than in normal BM (Figure S2A). It has also been reported that Ki-67 expression is increased in cells with activated SGOC pathway and decreased in cells with inhibited SGOC pathway [28]. We examined the expression levels of Ki-67 in normal BM and BM-MDSC using flow cytometry. We observed that Ki-67 expression was increased in BM-MDSCs, indicating that BM-MDSCs proliferate more rapidly than normal BM cells (Figure S2B). In summary, we ascertained that the intracellular concentration of SGOC inputs is heightened, given that the expression levels of *Phgdh* mRNA and Ki-67 are increased in BM-MDSC compared to normal BM. Studies will need to further investigate the potential of the SGOC pathway of differentiating MDSCs as a therapeutic target. PCs account for 50% of cellular phospholipids and are the major components of cell membranes. PEs are the second most frequently detected type of phospholipid in cell membranes after PC. In addition to cell membranes, PE is also present in mitochondrial membranes and plays a crucial role in the growth and stability of mitochondria [29]. In SPL-MDSCs, PE was decreased while PC was increased, which can be presumed to be associated with mitochondrial dysfunction. It was found that an increased intracellular PC/PE ratio caused by a decrease in mitochondrial PE impairs cell survival and growth, accompanied by a reduction in ATP production, cellular ATP level, and oxygen consumption rate [30]. Conversely, a reduction in the PC/PE ratio increases mitochondrial respiration [31]. The increase in the PC/PE ratio in SPL-MDSCs implies that the mitochondrial function of SPL-MDSCs might be decreased.

De novo lipogenesis (DNL) is a metabolic pathway that converts pyruvate produced during glycolysis into fatty acids. Pyruvate is transported into mitochondria and sequentially metabolized to acetyl-CoA and citric acid. Citric acid is then transferred to the cytosol and converted into palmitic acid (C16:0), which is the primary product of DNL [32]. Among palmitic acid-containing phospholipids, the major products of DNL are PC and PE 16:0/18:2, 16:0/18:1, and 16:0/22:6 [33]. In our experiment, SPL-MDSCs had lower PE 16:0/18:2, plasmenyl-PE 16:0/18:1, and plasmenyl-PE 16:0/18:2 concentrations compared to other cell types. In addition, other palmitic acid-containing phospholipids, namely PI 16:0/18:2, PI 16:0/20:4, and plasmenyl-PE 18:0/16:0, showed higher concentrations in BM-MDSCs compared to SPL-MDSCs. Interestingly, while glucose, the main substrate of DNL, was elevated during the differentiation of MDSCs, palmitic acid, the major product of DNL, was decreased in SPL-MDSC. Therefore, the high PC/PE ratio observed in this study suggests that DNL might be suppressed in SPL-MDSCs due to enzymatic defects or mitochondrial dysfunction.

Considering the VIP values of the 75 metabolites and lipids detected, the top ten consisted of five metabolites and five lipids that had the highest VIP values. For the five lipids in the top ten, PS 18:0/20:3 and PI 18:1/18:2 were decreased in SPL-MDSCs compared to normal BM, while PC 18:1/18:2, PC 18:0/18:0, and PG 16:0/18:1 were increased in SPL-MDSCs compared to normal BM. Meanwhile, the five metabolites in the top ten were putrescine, glycine, glutamic acids, glucose, and G6P.

Putrescine is a diamine, metabolized sequentially from arginine and ornithine, that is required by mammalian cells for growth and proliferation [34]. Arginase, which metabolizes arginine, is the limiting factor for putrescine synthesis in myeloid cells [35]. MDSCs are well known for having high levels of arginase, which restricts the activation and growth of T cells [36]. Putrescine concentration is elevated in the BM of leukemia patients [37] and is also increased in diverse types of proliferating cells [38]. BM-MDSCs are immature, proliferating myeloid cells that are abundantly produced and accumulated in the BM. In our experiment, the highest concentration of putrescine was found in BM-MDSCs, confirming that BM-MDSCs also require putrescine for growth and proliferation.

Other metabolites with high VIP ranking are amino acids such as glycine and glutamic acid, which are used as building blocks in proliferating cells [39]. These amino acids are commonly increased in BM-MDSCs compared to normal BM. In addition, serine and aspartic acid, which are used in nucleotide synthesis and cell proliferation, were also increased in BM-MDSCs compared to normal BM. Tryptophan showed the lowest concentration in SPL-MDSCs; this might be due to the overexpression of IDO, which catabolizes tryptophan into kynurenine in MDSCs [40].

Glucose is a starting material for glycolysis, which plays a pivotal role in intracellular metabolism [41]. It is not only a source of ATP; glucose also plays an important role in interconnecting the intricate metabolic processes within cells through the generation of various metabolic intermediates during glycolysis. Glucose is metabolized to G6P by phosphorylation and is involved in numerous metabolic pathways, such as glycolysis, glycogenesis, the pentose phosphate pathway, and the tricarboxylic acid cycle [42,43]. Therefore, maintaining an appropriate concentration of glucose in cells is very important. Once transported into cells, glucose is immediately phosphorylated into G6P by HK to prevent loss caused by outward glucose diffusion. Our results showed that the intracellular glucose concentration was increased 9-fold as normal BM cells differentiated into BM-MDSCs, and 20-fold as they differentiated into SPL-MDSCs. Even in the case of 3T3-L1 cells, which can differentiate into various cell types, the difference in intracellular glucose concentration between fibroblasts and adipocytes is only five-fold [44]. Considering that glucose is essential for maintaining cellular function and cell metabolism, the massive rise in glucose concentration observed in SPL-MDSCs is not a common phenomenon. Our data showed that overexpressed GLUT1 in MDSCs transports excess glucose into the MDSCs, creating a high-glucose environment inside the MDSCs. In addition, the data also suggest that GLUT1 overexpression is a unique feature of MDSCs, and GLUT1 is downregulated by the terminal maturation of MDSCs. The experimental results robustly support the previous finding that MDSCs favor aerobic glycolysis over OXPHOS [45]. The decrease in cellular ATP production due to the preference for glycolysis must be compensated for by consuming large amounts of glucose. Several studies showed the importance of glucose concentration and metabolic processes in MDSCs. It has been demonstrated that treatment with 2-Deoxy-D-glucose (2-DG), a glycolysis inhibitor in the early stages of glycolysis, suppresses the generation and function of MDSCs [45,46]. Therefore, our results indicate that the high concentration of glucose within MDSCs is essential for generating necessary energy and metabolites through glycolysis, which in turn is critical for maintaining the survival and function of MDSCs. On the contrary, it has been reported that the frequency of MDSCs increases in animal diabetes models and in diabetic patients [47]. In addition, a recent study has revealed that a hyperglycemic environment promotes the differentiation of BM cells into MDSCs and enhances their immunosuppressive function [48]. In summary, MDSCs prefer a hyperglycemic environment and acquire glucose through GLUT1 overexpression, thus creating an intracellular hyperglycemic environment. The metabolic process utilizing abundant intracellular glucose is essential for the survival of MDSCs.

In summary, we found that MDSCs exhibit abnormal glucose metabolism compared to normal cells throughout their differentiation process, likely due to MDSC-specific overexpression of GLUT1. In addition, we observed lipid changes in SPL-MDSCs, suggesting impaired mitochondrial function. Thus, it is expected that MDSCs prefer to generate ATP through mitochondria-independent aerobic glycolysis. Numerous therapeutic strategies targeting MDSCs are currently being investigated. These include strategies aimed at MDSC maturation, blockade of MDSC accumulation, inhibition of MDSC immunosuppression, and depletion of MDSCs [49]. Our study investigated changes in metabolites and lipids during MDSC differentiation and found that the SGOC pathway and glucose metabolism were notably altered in MDSCs compared to normal BM. There is a potential for developing MDSC inhibitors that target these changes, thus modulating the immune-suppressive tumor microenvironment and improving the effectiveness of anticancer immunotherapy.

## 5. Conclusions

In this study, we conducted a comprehensive metabolomic and lipidomic analysis of MDSCs, identifying 25 metabolites and 50 lipids. It was confirmed that levels of various biomolecules change as cells differentiate from BM precursors to BM-MDSCs and SPL-MDSCs. BM-MDSCs showed increased inputs for the SGOC pathway compared to normal BM, while SPL-MDSCs showed altered metabolomic and lipidomic profiles related to mitochondrial dysfunction. The glucose concentration in MDSCs was significantly enhanced as MDSCs differentiated, and the increase in glucose was due to increased GLUT1 levels, mediating the influx of glucose into cells. Further studies focusing on the SGOC pathway during MDSC development and glycolysis in mature MDSCs are required.

## Figures and Tables

**Table 1:** Metabolites and lipids identified with variable influence on projection (VIP) values greater than 1.0.

No.	Compound	VIP Values
1	Putrescine	1.561
2	Glucose-6-phosphate	1.473
3	PS 18:0/20:3	1.372
4	PI 18:1/18:2	1.364
5	PC 18:1/18:2	1.350
6	Glucose	1.337
7	PC 18:0/18:0	1.314
8	PG 16:0/18:1	1.284
9	Glycine	1.283
10	Glutamic acid	1.245
11	PC 18:2/20:4	1.223
12	PS 16:0/18:2	1.215
13	Plasmenyl PE 16:0/18:2	1.211
14	PC 18:2/20:0	1.192
15	Plasmenyl PE 16:0/18:1	1.185
16	PI 16:0/20:4	1.177
17	PS 18:0/18:0	1.163
18	PC 18:2/20:3	1.156
19	PS 18:0/22:6	1.151
20	PS 18:1/20:4	1.148
21	Tryptophan	1.132
22	Aspartic acid	1.108
23	PC 18:2/18:2	1.100
24	PI 16:0/18:2	1.095
25	Plasmenyl PE 18:0/16:0	1.030
26	Cer 18:1/18:0	1.028
27	PS 18:1/18:2	1.026
28	Plasmenyl PE 18:0/20:5	1.026
29	Uric acid	1.018
30	PS 16:0/18:0	1.014
31	Maltose	1.009
32	Myo-inositol	1.005

## Data Availability

The data presented in this study are available on request from the corresponding author. Data is not publicly available due to privacy.

## Supplementary Materials

The following supporting information can be downloaded at: https://www.mdpi.com/article/10.3390/metabo13040477/s1, Figure S1. Purity of sorted cells. CD11b^+^GR-1^+^ cells from bone marrow and spleen were sorted using a cytometer. Prior to sorting, CD11b^+^ splenocyte were positively selected using magnetic beads. Figure S2. mRNA and protein expression level related to SGOC pathway. Normal BM and BM-MDSC were harvested from normal and tumor-engrafted mice. (A) The *Phgdh* mRNA expression levels were measured by qRT-PCR and normalized by *Actb*. (B) Ki-67 expression levels of CD11b^+^Gr-1^+^ cells were measured by flow cytometer. Data shown are expressed as mean ± SEM of three replicates with similar results. *, *p* < 0.05. The *p* values were obtained by Student’s *t*-test.
