# Supplementary material for: Discovery of Myeloid-Derived Suppressor Cell-Specific Metabolism by Metabolomic and Lipidomic Profiling
Source: Metabolites. 2023 Mar 27;13(4):477. doi: 10.3390/metabo13040477 (PMC10147080; doi:10.3390/metabo13040477)
Supplement: Supplementary file 1 [file metabolites-13-00477-s001.zip › metabolites-2251079-supplementary.pdf]

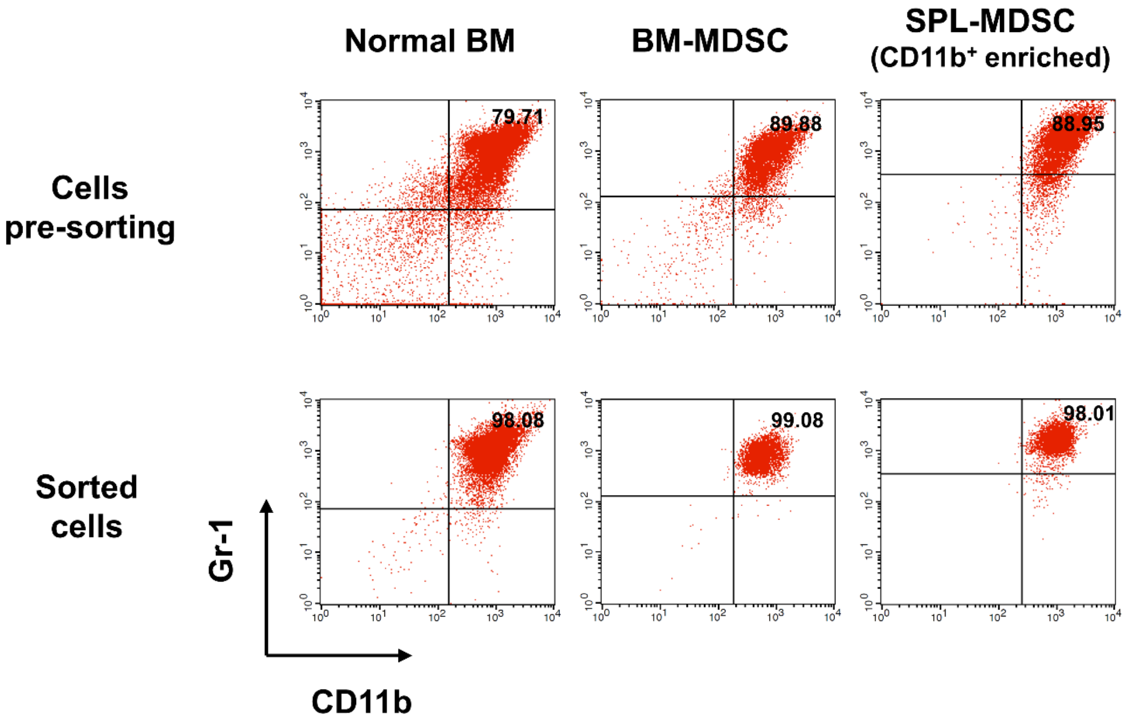

Figure S1. Purity of sorted cells. CD11b$^+$GR-1$^+$ cells from bone marrow and spleen were sorted using a cytometer. Prior to sorting, CD11b$^+$ splenocyte were positively selected using magnetic beads.

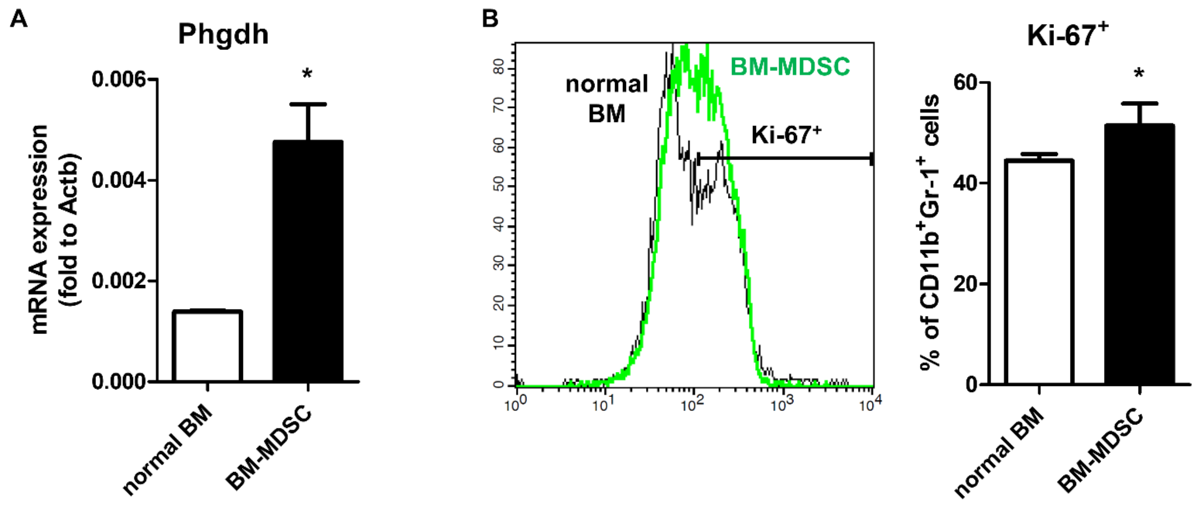

Figure S2. mRNA and protein expression level related to SGOC pathway. Normal BM and BM-MDSC were harvested from normal and tumor-engrafted mice. (A) The *Phgdh* mRNA expression levels were measured by qRT-PCR and normalized by *Actb*. (B) Ki-67 expression levels of $CD11b^+Gr\text{-}1^+$ cells were measured by flow cytometer. Data shown are expressed as mean ± SEM of three replicates with similar results. *, $p < 0.05$. The p values were obtained by Student's t-test.
